# Hémosidérose pulmonaire révélant un déficit en HLA classe II chez un nourrisson de 9 mois: à propos d'un cas

**DOI:** 10.11604/pamj.2026.53.33.50573

**Published:** 2026-01-26

**Authors:** Lemrabott Hamada Beddi, Amale Hassani, Brahim Elhasbaoui, Abdelilah Radi, Abdelhakim Ourrai, Rachid Abilkassem, Agader Aomar

**Affiliations:** 1 Service de Pédiatrie Générale, Hôpital Militaire d'Instruction Mohamed V, CHU Ibn Sina, Rabat, Maroc

**Keywords:** Hémosidérose, déficit en HLA classe 2, enfant, cas clinique, Pulmonary hemosiderosis, class II HLA deficiency, children, case report

## Abstract

L'hémosidérose pulmonaire est une affection respiratoire rare et sévère chez l'enfant correspondant à une hémorragie alvéolaire chronique. Les déficits immunitaires combinés sont caractérisés par un défaut quantitatif ou qualitatif des lymphocytes T, associé ou non à un déficit complet ou partiel de synthèse d'immunoglobulines. Nous rapportons une association rare d'hémosidérose pulmonaire et d'un déficit en HLA classe II, chez le nourrisson T, âgé de 9 mois, admis pour détresse respiratoire aiguë et pâleur. L'examen clinique trouvait une pâleur cutanéo-muqueuse, une saturation artérielle en oxygène à 87%, une fréquence respiratoire à 65 cycles/min, avec des râles crépitants diffus aux deux hémichamps pulmonaires. L'hémogramme objectivait une anémie normochrome normocytaire régénérative et une neutropénie. Par ailleurs, on notait une CRP à 59 mg/L, un test de Coombs direct positif, une ferritine élevée à 1095 ng/mL. La TDM thoracique montrait un aspect en faveur d'une pneumopathie interstitielle bilatérale. L'étude cytologique du lavage broncho-alvéolaire a objectivé un liquide hémorragique, avec à l'étude cytologique des sidérophages à 46%, une coloration de Perls positive et un score de Gold à 187. Le bilan immunitaire a permis de poser le diagnostic de déficit immunitaire combiné à un déficit en HLA classe II. T a été mis sous antibiothérapie, deux transfusions de culots globulaires, une corticothérapie générale, des nébulisations par le salbutamol et le Pulmicort, et une perfusion d'immunoglobulines polyvalentes. L'évolution était favorable. Un traitement par allogreffe de cellules souches hématopoïétiques est en cours. Cette observation rappelle l'importance de mettre en cause le diagnostic de bronchiolite aiguë, notamment lorsque l'évolution semble atypique et inhabituelle.

## Abstract (en)

Pulmonary hemosiderosis is a rare and severe respiratory condition in children corresponding to chronic alveolar haemorrhage. Combined immune deficiencies are considered rare diseases, characterised by a quantitative or qualitative defect of T lymphocytes, associated or not with a complete or partial deficiency of immunoglobulin synthesis. We report a rare association of pulmonary hemosiderosis and class II HLA deficiency. Infant T, aged 9 months, was admitted for acute respiratory distress and pallor. The clinical examination found a marked mucocutaneous pallor, arterial oxygen saturation at 87%, a respiratory rate at 65 cycles/min, with diffuse crackling rales at both pulmonary hemifields. The blood count objectified with regenerative normocytic normochrome anemia and neutropenia. In addition, there was a CRP at 59 mg/l, a positive direct Coombs test, and a ferritin level elevated to 1095 ng/ml. Chest CT showed an aspect in favor of severe bilateral interstitial lung disease. The cytological study of bronchoalveolar lavage objectified a hemorrhagic fluid, with the cytological study siderophage at 46%, a positive Perls staining and a Gold score of 187. The immune assessment made it possible to make the diagnosis of combined immune deficiencies by HLA class II deficiency. T was put on antibiotic therapy, two blood cell transfusions, general corticosteroid therapy, nebulizations of salbutamol, Pulmicort, and an infusion of versatile immunoglobulins. The evolution was favorable. Treatment with allogeneic hematopoietic stem cell transplantation is ongoing. This observation recalls the importance of questioning the diagnosis of acute bronchiolitis, especially when the evolution seems atypical and unusual.

## Introduction

L'hémosidérose pulmonaire est une affection respiratoire rare et sévère chez l'enfant correspondant à une hémorragie alvéolaire chronique et/ou à des exacerbations aiguës. La présentation classique de l'Hémosidérose Pulmonaire Idiopathique (HPI) comprend une hémoptysie, une anémie et des opacités alvéolaires radiologiques [1]. Nous rapportons ici un cas démonstratif, révélateur d'une pathologie rare et grave: le défaut d'expression des molécules HLA de classe II. Il s'agit d'une maladie génétique rare, à transmission autosomique récessive, liée à des mutations des gènes régulant l'expression des molécules HLA de classe II [2].

## Patient et observation

**Information du patient:** T. nourrisson de sexe masculin, âgé de 9 mois, admis pour une détresse respiratoire aiguë et pâleur.

**Chronologie:** le nourrisson avait dans les antécédents personnels une notion d'hospitalisation à l'âge de 7 mois pour bronchiolite aiguë, premier épisode. Dans les antécédents familiaux, on notait une consanguinité de premier degré, une mère asthmatique et deux décès dans la fratrie, une sœur à l'âge de 6 mois dans un tableau de détresse respiratoire et un frère à l'âge de deux mois de cause inconnue. La grossesse s'était déroulée sans particularité avec une naissance au terme de 37 semaines d'aménorrhée par césarienne dont l'indication était une présentation de siège, avec un poids de naissance de 3 500 g, un Apgar de 10/10 et une bonne adaptation à la vie extra-utérine. Le développement psychomoteur s'avérait normal et l'allaitement était mixte avec une diversification alimentaire à l'âge de 6 mois.

**Résultats cliniques:** l'examen clinique trouvait à l'admission un nourrisson hypotonique, hyporéactif, en mauvais état général, fébrile à 38,5°C avec une fréquence cardiaque à 130 battements/min, une fréquence respiratoire à 65 cycles/min, une saturation artérielle en oxygène à 87% à l’air ambiant, un TRC < 3 s, un poids à 9 kg (+1 DS), une taille à 71 cm (+1 DS). Par ailleurs, on notait une pâleur cutanéo-muqueuse marquée, un tirage sous-costal, un battement des ailes du nez et des râles crépitants diffuses aux deux hémichamps pulmonaires. L'auscultation cardiaque était normale, sans souffle ni galop ; les aires ganglionnaires étaient libres; il n'y avait pas d'hépato-splénomégalie ni de signes cutanés.

**Figure 1:**
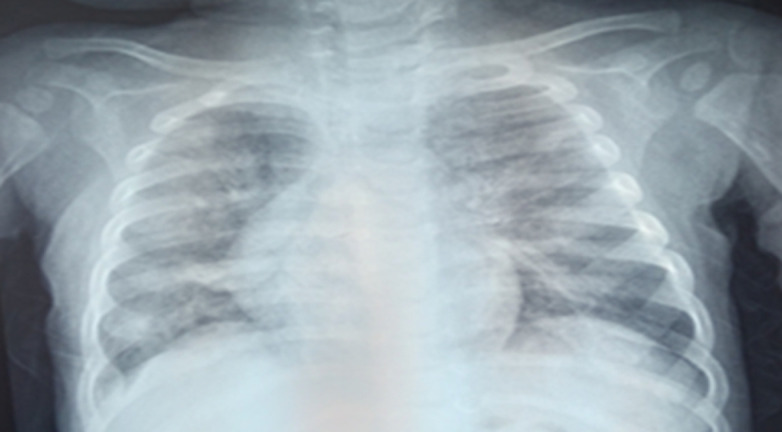
radiographie du thorax montrant des opacités alvéolo-interstitielles bilatérales

**Figure 2:**
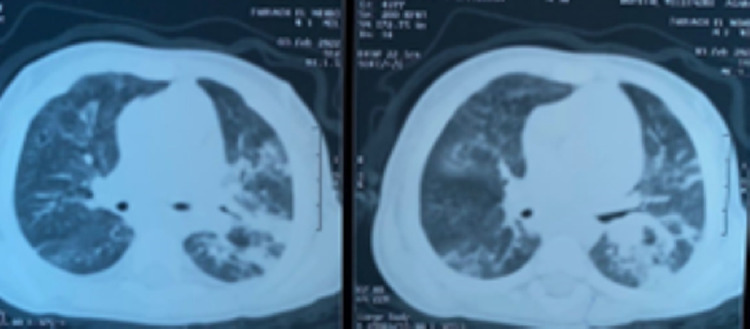
TDM thoracique montrant un aspect de pneumopathie interstitielle bilatérale sévère

**Figure 3:**
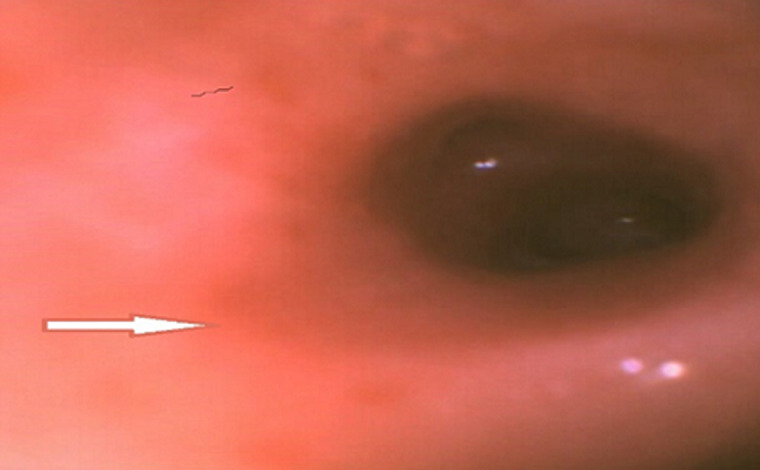
aspect très inflammatoire de la muqueuse bronchique lors du LBA

**Figure 4:**
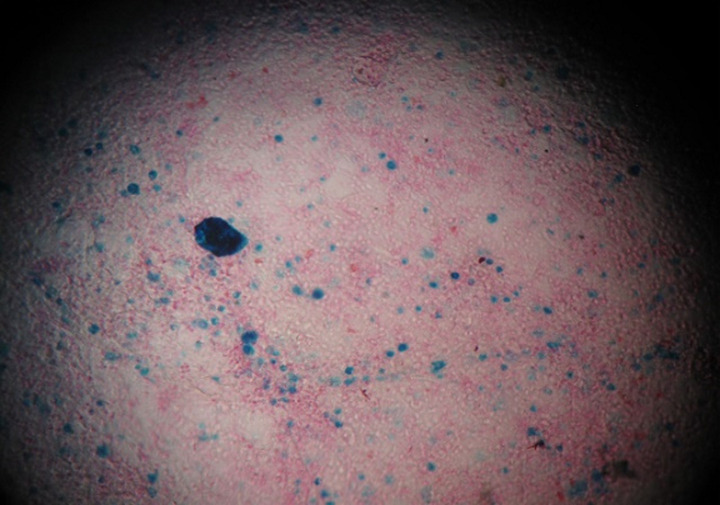
coloration de Perls avec contenu des macrophages alvéolaires en bleu, intensité de la coloration fonction de la charge en hémosidérine

**Démarche diagnostique:** la radiographie du thorax de face mettait en évidence un syndrome alvéolo-interstitiel diffus (Figure 1). Le diagnostic initialement retenu était celui d'une bronchiolite aiguë, deuxième épisode. Une hospitalisation était décidée avec une oxygénothérapie à 3 L/min, des nébulisations répétées au salbutamol et du méthylprednisolone par voie intraveineuse à raison de 2 mg/kg toutes les 8 heures. L'hémogramme montrait des leucocytes à 5800/mm^3^, des lymphocytes à 4200/mm^3^, une neutropénie sévère à 200/mm^3^, une anémie à 7,1 g/dl normochrome normocytaire. La CRP était à 35 mg/l et le bilan infectieux (sérologie SARS-CoV-2 avec IgG et IgM, PCR SARS-CoV-2, ECBU) était négatif. Devant l'absence d'amélioration clinique franche avec persistance de la détresse respiratoire hypoxémiante et l'aggravation du syndrome anémique, une TDM thoracique était demandée. L'échographie cardiaque était normale. Cette présentation clinique de bronchiolite était atypique et il s'agissait finalement d'une pneumopathie infiltrative diffuse vu l'aspect à la TDM thoracique (Figure 2). Une analyse sémiologique des données de l'interrogatoire, à savoir la consanguinité parentale de premier degré et l'antécédent de deux décès dans la fratrie survenus avant l'âge d'un an, a permis d'attirer l'attention. L'auscultation pulmonaire trouvait paradoxalement des fins râles sibilants contrastant avec la sévérité de la détresse respiratoire, ceci pouvant s'expliquer par l'anémie qui semblait être au-devant du tableau. Devant cette pneumopathie infiltrative diffuse, nous avons évoqué d'autres diagnostics étiologiques. En effet, il est important d'évoquer une atteinte pulmonaire dans le cadre d'une maladie générale (collagénoses, vascularites, sarcoïdose, maladies de surcharge, histiocytose langhéransienne, infiltrations tumorales, syndromes lymphoprolifératifs, hémosidérose pulmonaire, tuberculose pulmonaire, infections opportunistes sur déficit immunitaire héréditaire ou acquis…). Les examens microbiologiques réalisés (PCR multiplex sur prélèvement nasal à la recherche du VRS, virus influenza, parainfluenza et adénovirus, recherche de *Pneumocystis jirovecii* dans les aspirations bronchiques, sérologie contre le cytomégalovirus) sont négatifs et le dosage de l'enzyme de conversion était normal. Devant les éléments atypiques précédemment décrits, le syndrome pneumo-anémique et le syndrome alvéolo-interstitiel bilatéral. Un lavage broncho-alvéolaire était réalisé, montrant un aspect très inflammatoire de la muqueuse bronchique (Figure 3). L'étude cytologique trouvait des macrophages à 46%, des lymphocytes à 12%, PNN à 42%, une coloration de Perls positive, avec un score de Golde estimé à 187 (Figure 4). Une telle présentation doit évoquer une infection opportuniste sur un déficit immunitaire acquis ou primitif, ce qui a imposé de s'interroger sur le statut immunitaire de notre patient. Le bilan de première intention dans ce contexte comprend un hémogramme, une sérologie VIH, un dosage pondéral des immunoglobulines et un immunophénotypage lymphocytaire. L'hémogramme a révélé une anémie et une neutropénie. Le dosage pondéral des immunoglobulines a mis en évidence un déficit en IgA. L'immunophénotypage lymphocytaire a précisé une proportion diminuée de lymphocytes CD3 et CD4 natifs et l'absence d'expression des molécules HLA de classe 2 sur les lymphocytes B et les monocytes. La recherche sur prélèvement de LBA d'un *Pneumocystis jirovecii* et d'un cytomégalovirus était négative. Le diagnostic de pneumopathie interstitielle diffuse a donc conduit à la découverte d'un déficit immunitaire combiné (déficit en HLA de classe 2) chez ce nourrisson.

**Intervention thérapeutique:** le patient a été pris en charge aux urgences. Traitement de l'hémosidérose pulmonaire: ce nourrisson a reçu une corticothérapie à base de prednisone à la posologie de 2 mg/kg/j, associée à un traitement adjuvant. Prise en charge du déficit immunitaire: elle consistait en une perfusion d'immunoglobulines polyvalentes tous les 21 jours. Cependant, en attendant de trouver un donneur compatible, une allogreffe de cellules souches hématopoïétiques constitue le traitement de référence chez notre patient. Une antibioprophylaxie au cotrimoxazole à raison de 20 mg/kg/j a débuté chez notre patient afin de prévenir les infections opportunistes, en l'occurrence la pneumocystose.

**Suivi du patient:** après un recul de 4 mois, il est marqué par la survenue d'une allo-immunisation (RAI positifs) surajoutée à l'anémie hémolytique auto-immune préexistante ayant comme conséquence des hémolyses aiguës répétées nécessitant des transfusions sanguines itératives à un rythme de 3 semaines. Cependant, sur le plan respiratoire, le nourrisson n'a plus de symptomatologie respiratoire.

**Perspective du patient:** notre patient est en attente d'une greffe de cellules souches hématopoïétiques.

**Consentement du patient:** les parents ont donné leur consentement éclairé pour la publication de ce cas clinique et des images associées. Ils ont été informés que toutes les données permettant l'identification de leur enfant ont été supprimées afin de garantir l'anonymat.

## Discussion

L'hémosidérose pulmonaire est une pathologie rare mais grave. Une détresse respiratoire aiguë sifflante chez un nourrisson est fréquemment une bronchiolite aiguë [2]. Dans notre observation, plusieurs éléments atypiques ont fait douter de ce diagnostic: l'absence d'amélioration clinique franche après un traitement symptomatique bien conduit, le syndrome anémique associé et le syndrome alvéolo-interstitiel radiologique marqué, bien que ce dernier élément puisse s'associer à un tableau clinique de bronchiolite et à d'authentiques pneumonies virales (VRS, adénovirus, influenza et parainfluenza) [3]. En effet, certains signes d'alerte (les cytopénies chez notre patient) doivent être recherchés et orientés vers l'éventualité d'autres diagnostics tels qu'une pathologie respiratoire chronique (pneumopathies interstitielles diffuses, fibroses pulmonaires, dysplasies broncho-pulmonaires, mucoviscidose), ou un déficit immunitaire primitif [2]. Ainsi, le tableau respiratoire évoquant une bronchiolite a révélé une pneumopathie infiltrative diffuse, qui est l'hémosidérose pulmonaire. L'incidence de l'HPI est d'environ 0,24 à 1,23 par million d'habitants et affecte principalement les enfants [4].

Chez notre patient, le délai diagnostique est de deux mois en raison de la symptomatologie parlante et de l'intolérance à cette hémorragie intra-alvéolaire. L'une des limites de notre observation est que le diagnostic d'HPI n'a pas été confirmé par une biopsie pulmonaire. Cependant, en présence de macrophages chargés d'hémosidérine dans le LBA et de symptômes pulmonaires chroniques, un diagnostic d'HPI peut être posé [5]. Les antécédents de deux décès inexpliqués dans la fratrie et le caractère récidivant ou persistant des symptômes nous ont poussés à rechercher un déficit immunitaire, plus précisément un déficit en HLA classe II. Dans cette pathologie, les premières infections apparaissent en moyenne vers l'âge de 4 mois. Les modes de révélation les plus fréquents sont les infections broncho-pulmonaires récidivantes et la diarrhée chronique [3]. Au plan immunologique, le déficit en HLA de classe II affecte les réponses immunitaires humorales (hypogammaglobulinémie) et cellulaires spécifiques (lymphopénie CD4 et défaut de réponse aux stimulations antigéniques) [3]. Les molécules HLA de classe II sont des glycoprotéines hétérodimériques transmembranaires codées par des gènes situés sur le bras court du chromosome 6 [3]. La majorité des patients atteints est d'origine maghrébine [6]. Les données de la littérature montrent que l'hémosidérose pulmonaire est plutôt fréquemment associée à l'intolérance aux protéines de lait de vache (syndrome de Heiner), à la maladie cœliaque (syndrome de Lane-Hamilton), ainsi qu'à la trisomie 21 [7]. Bien qu'elle soit extrêmement rare, aucun cas n'a été rapporté dans la littérature. L'association d'une hémosidérose pulmonaire et d'un déficit immunitaire par défaut d'expression des molécules HLA de classe II conforte l'hypothèse d'origine immunologique commune aux deux affections. Les manifestations auto-immunes associées aux déficits immunitaires primitifs décrites dans la littérature étaient une anémie hémolytique auto-immune, une thrombopénie, une neutropénie et une vascularite multiviscérale [8]. L'intérêt de notre observation était de rapporter une manifestation auto-immune inhabituelle et urgente (hémosidérose pulmonaire) ayant révélé le déficit immunitaire en HLA classe II chez notre patient. L'évolution de l'HPI est très variable d'un enfant à l'autre. La maladie peut évoluer lentement et provoquer une anémie chronique et une insuffisance respiratoire progressive (par fibrose pulmonaire). Dans les cas d'hémosidérose pulmonaire secondaire, l'évolution dépend de la maladie à l'origine de l'hémosidérose, pour laquelle il peut y avoir un traitement adapté. Si la greffe des cellules souches hématopoïétiques permet une rémission de l'hémosidérose pulmonaire chez notre patient, une origine secondaire au déficit immunitaire en HLA classe II saurait, dans ce cas très probable, être l'objectif d'études scientifiques ultérieures.

Une étude indienne prometteuse publiée en 2019 démontre que tous les patients se sont vus prescrire un régime sans lait et ont été traités par prednisolone orale (1-1,5 mg/kg/jour) et hydroxychloroquine (HCQ). Les patients ayant présenté une récidive d'hémorragie pulmonaire lors de la diminution des stéroïdes ont été traités par l'azathioprine, qui a ensuite été prescrite systématiquement après 2 à 4 semaines de traitement, lorsque la diminution progressive des stéroïdes a été amorcée. Un enfant qui ne répondait pas à l'azathioprine a été induit par des impulsions mensuelles de cyclophosphamide par voie intraveineuse. Dans cette série de patients, les résultats étaient satisfaisants. En effet, lors du suivi (durée moyenne de 3 ans, 10 mois), il n'y a pas eu de récidive et, après une rémission de plus de deux ans, l'azathioprine a été progressivement réduite et l'hydroxychloroquine a été poursuivie [9]. Le pronostic de l'hémosidérose pulmonaire semble s'être amélioré avec le temps. Alors qu'il y a vingt ans, la survie moyenne était de trois ans après le diagnostic, les données récentes montrent une survie à cinq ans dans 86% des cas [6]. Cette amélioration significative est probablement due à l'instauration précoce et à l'utilisation à long terme d'un traitement immunosuppresseur. Pour se résumer, la corticothérapie représente la première ligne de traitement, y compris le palmitate de dexaméthasone incorporé dans les liposomes (lipostéroïde). La transplantation de cellules souches hématopoïétiques en cas de déficit en CMH de classe II est difficile du fait des complications, notamment la toxicité liée au traitement, les infections opportunistes graves, le rejet du greffon, même dans le contexte d'un greffon de frère ou sœur HLA apparié [10]. Nos données combinées à la littérature publiée suggèrent que l'utilisation de busulfan associé au cyclophosphamide est insuffisante pour permettre une prise de greffe reproductible chez les patients atteints de cette maladie [10].

## Conclusion

Cette observation rappelle l'importance de mettre en cause le diagnostic de bronchiolite aiguë, notamment lorsque l'évolution semble atypique et inhabituelle. La vigilance du clinicien doit être constante afin d'évoquer rapidement un diagnostic différentiel, dont l'évolution peut être fatale en l'absence de traitement. En effet, un premier épisode de dyspnée sévère chez un nourrisson peut être le mode de révélation d'une pathologie dysimmunitaire. Le pronostic, certes sombre, peut être amélioré par une prise en charge étiologique rapide et précoce, la corticothérapie générale et l'allogreffe de cellules souches hématopoïétiques.
